# Success rate of infrazygomatic miniscrews considering their design and insertion techniques. A review

**DOI:** 10.21142/2523-2754-1003-2022-117

**Published:** 2022-09-28

**Authors:** María Emely Rodríguez-Rimachi, Mónica Ivette Malpartida-Pacheco, Walter Carlos Olazábal-Martínez

**Affiliations:** 1 School of Dentistry, Andina del Cusco University, Cusco, Peru. maremy55@gmail.com Universidad Andina del Cusco School of Dentistry Andina del Cusco University Cusco Peru maremy55@gmail.com; 2 School of Dentistry, San Martin de Porres University, Lima, Peru. monica.mp25@gmail.com Universidad de San Martín de Porres School of Dentistry San Martin de Porres University Lima Peru monica.mp25@gmail.com; 3 School of Dentistry, Inca Garcilazo de la Vega University, Lima, Peru. wal.olazabal@gmail.com Universidad Inca Garcilaso de la Vega School of Dentistry Inca Garcilazo de la Vega University Lima Peru wal.olazabal@gmail.com

**Keywords:** miniscrew, infrazygomatic, success rate, minitornillos, infracigomático, tasa de éxito

## Abstract

Miniscrews offer the possibility of performing dental movements, minimizing unwanted side effects and enhancing effectiveness. Extra-alveolar miniscrews are ideal as they provide excellent primary stability and avoid anatomical structures. However, in some cases the primary stability is lost before achieving the success of the mechanics used and thus, the most likely causes of failure should be determined. The purpose of this review was to analyze the success rate of infrazygomatic miniscrews, considering their design and the insertion techniques used. Data collection of this literature review was carried out by searching PubMed, Wiley, Google Scholar sites, SCIELO, Elsevier and Dialnet for publication made from 2003 to June 2022. The search was carried out on June 10^th^, 2022 and the following keywords were used; infrazygomatic crest, miniscrews, anchorage and stability. Different topics were analyzed and discussed highlighting their clinical relevance. After analyzing the 798 articles, 566 were excluded. The remaining articles were re-analyzed and 153 articles were excluded for the title or abstract and 33 articles were excluded for the methodology. Finally, 46 items remained. After thoroughly analyzing all the articles included, this study concluded that the alloy of the miniscrew (stainless steel or titanium), perforation of the maxillary sinus and the placement area (adhered mucosa or mobile mucosa) do not influence the survival of the miniscrew. The evidence also indicates that the percentage of failure is lower in infrazygomatic compared to intraradicular miniscrews. Orthodontists can confidently and safely include infrazygomatic miniscrew in different orthodontic procedures.

## RESUMEN

Los minitornillos ofrecen la posibilidad de realizar movimientos dentales, lo que minimiza los efectos secundarios no deseados y potencia la eficacia. Los minitornillos extraalveolares son ideales ya que proporcionan una excelente estabilidad primaria y evitan estructuras anatómicas. Sin embargo, en algunos casos se pierde la estabilidad primaria antes de lograr el éxito de la mecánica utilizada y, por lo tanto, se deben determinar las causas más probables de la falla. El propósito de esta revisión fue analizar la tasa de éxito de los minitornillos infracigomáticos, considerando su diseño y las técnicas de inserción utilizadas. La recolección de datos de esta revisión bibliográfica se realizó mediante la búsqueda en PubMed, Wiley, sitios de Google Scholar, SciELO, Elsevier y Dialnet para publicaciones realizadas desde 2003 hasta junio de 2022. La búsqueda se realizó el 10 de junio de 2022 y se utilizaron las siguientes palabras clave: *cresta infracigomática, minitornillos, anclaje* y *estabilidad*. Se analizaron y discutieron diferentes temas destacando su relevancia clínica. Tras analizar los 798 artículos, se excluyeron 566. Los restantes fueron reanalizados y 153 resultaron excluidos por el título o resumen, y 33 por la metodología. Finalmente, quedaron 46 artículos. Tras analizar exhaustivamente todos los artículos incluidos, este estudio concluye que la aleación del minitornillo (acero inoxidable o titanio), la perforación del seno maxilar y la zona de colocación (mucosa adherida o mucosa móvil) no influyen en la supervivencia del minitornillo. La evidencia también indica que el porcentaje de fracaso es menor en los minitornillos infracigomáticos en comparación con los intrarradiculares. Los ortodoncistas pueden incluir con confianza y seguridad minitornillos infracigomáticos en diferentes procedimientos de ortodoncia.

## INTRODUCTION

Orthodontic miniscrews offer the possibility of promoting tooth movements supported by fixed points, minimizing unwanted side effects, making treatments more efficient and predictable, reducing the need for patient collaboration and simplifying orthodontic biomechanics ^1^.

Miniscrews are easy to use, highly biocompatible and resistant to loads and corrosion. The peculiarity of these devices lies in the ability to establish primary stability without requiring osseointegration, which makes it easier and less invasive to remove the device. The insertion torque provides primary stability during miniscrew placement ^2^^-^^6^.

Miniscrew installation can be performed in different anatomical structures, including the hard palate, the mandibular buccal shelf (MBS) and the infrazygomatic crest (IZC) ^7^. Extra alveolar areas are distant to the dental roots ^8^^,^^9^, and generally have greater bone density, increasing primary stability. One commonly used extra alveolar region is the MBS, which may present anatomical variations in shape and size ^7^^,^^10^. In contrast, the most suitable extra alveolar area in the maxilla for miniscrew insertion is the IZC, located at the base of the eminence of the zygomatic crest, buccal to the roots of the maxillary molars ^11^.

It should also be taken into account that the thickness of the cortical bone increases in the apical direction, improving primary stability, and introducing a miniscrew apically at the mucogingival junction increases the possibility of success in cases of teeth with conical roots. When considering the ideal insertion zone in the vestibule, it should be taken into account that the attached mucosa in the maxillary molar area measures an average of 4 mm and a distance greater than this is needed and includes the mobile mucosa zone known as the "opportunity zone", in which the mucosa is fixed and attached to the periosteum and is considered non-mobile. 

Miniscrews can have different diameters, lengths, and body design and can be made of different alloys. Depending on the insertion technique, orthodontic miniscrews can be divided into self-drilling and self-tapping ^12^. It is reasonable to assume that different design features influence miniscrew stability and success rates. In particular, increased dimensions lead to increased contact with the bone surface ^13^.

In many articles the term "success" is synonymous with a high survival rate. Basically, miniscrews that meet the anchorage requirements for a given time are considered successful, while those that become mobile during treatment are considered failures. Consequently, the survival rate is a description of what percentage of miniscrews placed meet the anchorage requirements over time ^14^^,^^15^.

Many studies have investigated the failure rate of miniscrews in relation to different variables, such as gender, age, insertion site, loading latency, operator experience, and the physical characteristics of the screws. However, no study has identified all these variables in one study ^15^. Therefore, the aim of this review was to analyze the success rate of infrazygomatic miniscrews, considering their design and the insertion techniques.

## METHODOLOGY

### Information sources and search strategy

This study was based on the review of scientific articles found on the Internet in the main health databases. The search was conducted from April to June 10^th^, 2022, with no limitations as to the year of publication. The articles were similar in content terminology and focused on the problem. Virtual tools were used, starting with the US National Library of Medicine (MEDLINE via PubMed), Academic Google, Scielo, Wiley, Elsevier and Dialnet. The following search terms were used: miniscrew orthodontics, success, infrazygomatic and extra alveolar. Analytical and descriptive research as well as clinical trials and systematic reviews were included. (Table 1). 

**Table 1:** Strategies in the search for scientific articles in the main sources of information.

Source	Search Items
PubMed (183)	(((((orthodontics miniscrew) OR (microscrew)) OR (extra-alveolar miniscrew)) OR (infrazygomatic miniscrew)) AND (failure)) OR (success)
SciELO (19)	(orthodontic miniscrew) AND (orthodontic miniscrew) AND NOT (intraradicular)
Google Scholar (519)	(miniscrew orthodontics success) and (infrazygomatic miniscrew orthodontics)
Wiley (69)	"orthodontic miniscrew" anywhere and "infrazygomatic miniscrew" anywhere and "success" anywhere
Elsevier (6)	(miniscrew orthodontics) AND (miniscrew infrazygomatic) AND (failure)
Dialnet (2)	(orthodontic miniscrew) AND (infrazygomatic miniscrew) AND (success)

### Study selection

**Fig. 1:**
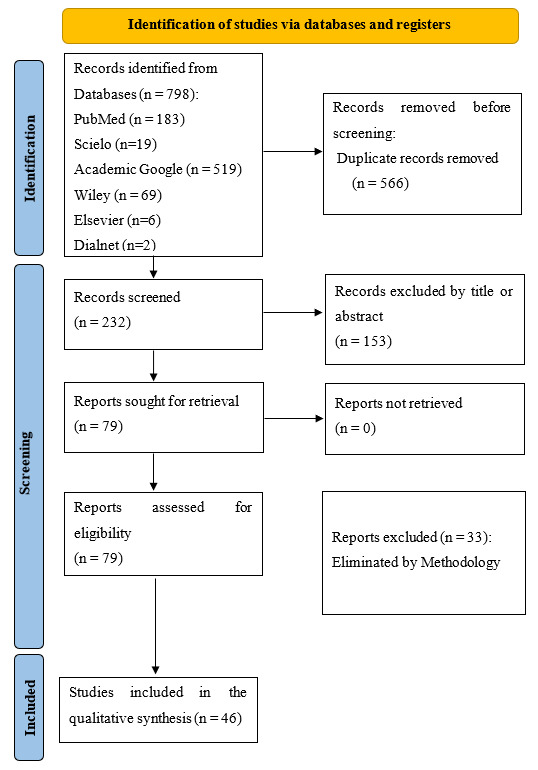
PRISMA. Flow diagram for scientific bibliographic search.

Finally, after a rigorous selection process, 46 articles were identified and met the following inclusion criteria: systematic revisions, observational studies, case reports, and randomized clinical trials in humans of both sexes. (Fig. 1).

## RESULTS AND DISCUSSION

### Design and main types of infrazygomatic miniscrews

In the maxilla, the IZC is one of the extra radicular sites for placement of temporary anchorage devices. It is used to provide anchorage for maxillary canine retraction, anterior retraction and intrusion of posterior upper teeth ^6^^,^^7^. Some studies also report that the IZC is a suitable anchorage site for insertion of a single miniscrew for corrections in the vertical dimension.6 It is reasonable to assume that different design features can influence the stability and success rates of miniscrews ^16^^-^^21^.

It is well known that an increase in the diameter of a miniscrew can lead to greater compromise of the bone surface, being the most important factor in terms of primary stability because an increase in diameter leads to greater torsional insertion ^(16, 22-24)^. This relationship is refuted by some studies and confirmed in others ^25^^-^^37^. Others have found a positive correlation between the length of miniscrew implants and the maximum possible load, which can be identified by primary stability ^35^^-^^37^. However, other authors report that the use of long miniscrews can cause microinjury to bone, and also emphasize the possibility of more frequent and more severe complications with the use of larger miniscrews ^38^^,^^39^.

Two main types of miniscrews are used for anchorage in routine orthodontic treatment: the self-drilling screw, which does not require the preparation of a pilot hole, and the self-tapping screw, which requires the drilling of a pilot hole, usually with a diameter smaller than that of the chosen screw. Several studies have compared the stability of these two types of screws. Many studies have found that self-drilling miniscrews provide better bone-to-screw contact and initial stability due to deeper thread and shear designs. In addition, it has also been described that these miniscrews take less time to insert and have a lower risk of root damage ^31^. However, other studies showed higher success rates with self-tapping screws, suggesting that pilot holes reduce the risk of microdamage to the bone and, thus, provide greater stability ^4^. Interestingly, a recent meta-analysis examining the literature comparing these two screw types found no significant difference between them ^32^. From the standpoint of stability, the choice of a screw type depends on clinician preference. 

### Extra-alveolar miniscrew insertion technique to ensure efficient biomechanics

In the bibliographic search, 72 articles related to appropriate insertion techniques were found, of which 5 were included because they were considered to provide the greatest contribution to the treated area and had greater methodological rigor.

**Fig. 2:**
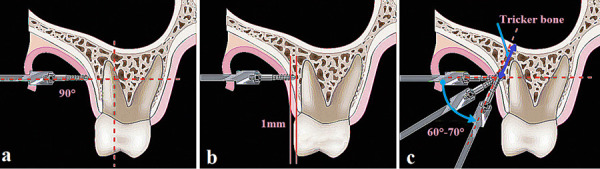
a. Initial insertion of the screw tip is perpendicular (90º) to the bone surface. b. The screw tip engages and penetrates a 1 mm bone cortex, buccal to the molar roots. c. After the miniscrew penetrates the outer layer of cortical bone, the screw driver is turned down 60° to 70°

In this sense, many authors agree that the appropriate technique for inserting miniscrews after administering local anesthesia is to drill with an explorer through the soft tissue to mark the desired skeletal site, which should be at the mucogingival junction or slightly apical to this. Then, the miniscrew should be directed perpendicularly towards the vestibular cortex, drilling 1 mm directly, and continue drilling while gradually turning until an angulation of between 60° and 70° is achieved to obtain a buccal insertion position to the roots of the molars ^35^^-^^38^. To avoid gingival hypertrophy, it is suggested that 5 mm of the miniscrew should overlap above the soft tissue. During the placement of miniscrews in the infrazygomatic crest, clinicians may have an “unwanted effect” such as maxillary sinus perforation; however, clinical findings have shown that sinus perforation itself does not significantly affect survival expectancy, especially when the insertion torque is adequate ^36^. (Fig. 2)

**Fig. 3:**
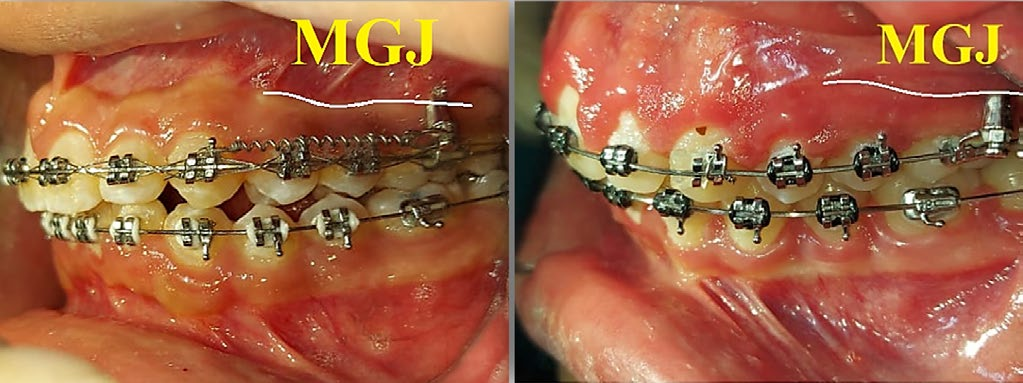
IZC Miniscrew placed in movable mucosa (left) and in attached gingiva (right). The broken white line is the mucogingival junction (MGJ).

Regarding the ideal insertion area in soft tissues, on average, the mucosa adhered to the maxillary molar area is 4 mm, and therefore, an area beyond this available distance is necessary to obtain an area of mobile mucosa known as the “opportunity zone”. In this area the mucosa is firmly attached to the periosteum and is considered non-mobile (Fig. 3). It should also be taken into account that the thickness of the cortical bone increases in the apical direction, and inserting a miniscrew apical to the mucogingival junction will greatly reduce the risk of contact with the root, which is a common factor in miniscrew failures ^37^.

In contrast, factors such as the location with respect to the occlusal plane and the angle of insertion are extremely important, with both usually being closely related to primary stability. In this regard, Liou *et al*. ^38^ evaluated 16 cone beam computed tomographies (CBCTs) to determine the area with the most favorable thickness of the IZC and found that it is located above the maxillary first molar (thickness from 5 mm to 9 mm). The same is found between 13 mm and 17 mm above the maxillary occlusal plane and can be reached directly by applying an angulation of 40° to 75°. Following of these references will prevent loss of anchorage and injury to the roots of the tooth and reduce irritation of the mucosa.

Murugesan *et al*. ^39^ evaluated 10 CBCTs taking the most desirable IZC thickness values using the distobuccal root of the maxillary first molar as a reference. They found that the widest thickness varied between 4.5 mm and 9 mm, and that this position is between 11 mm and 17 mm above the occlusal plane, at an angulation between 40° and 75°. It was concluded that in order to achieve insertion with adequate primary stability while also avoiding damage to the adjacent anatomical structures, a position at between 12 mm and 17 mm from the occlusal plane should be sought and an angle of 65 ° to 70 ° should be applied. The same year, these authors also analyzed 36 CBCTs to compare the dimension of the IZC in the different skeletal patterns and found that the thickness of the IZC varies between different skeletal patterns. However, they described that the thickness is more voluminous at the level of the mesiobuccal root of the upper second molar compared to the first molar, and suggested placing the miniscrew above this root.

Finally, Lima *et al*. ^40^ analyzed 86 CBCTs which were divided into three groups, according to hyperdivergent, hypodivergent and normodivergent facial biotypes. Six zones were measured between the second premolar and the distal root of the second molar at different heights: (5, 7, 9 and 11 mm) apical to the IZC and they found that in the three groups studied the most suitable area was located 11 mm from the alveolar crest between the first and second molars.

### Success rate and reasons for failure of extra alveolar miniscrews described in the main sources of scientific research

For the evaluation of this topic, 60 articles were found, 15 of which were selected for having the highest level of scientific evidence. The success of miniscrews is based on the anchorage achieved to perform the treatment while failure is considered when miniscrews show movement, fall out or need to be removed ^41^. To avoid complications, it is important to have good knowledge of the methodology and the most adequate site of insertion of the miniscrew. The risk of failure may be related to poor analysis of the insertion site, poor choice of equipment, poor treatment protocol, poor knowledge of orthodontic mechanics, or operator inexperience, and may also be related to infection by bacteria due to lack of adequate hygiene management. The absence of keratinized gingiva around the miniscrews increases the risk of inflammation, infection and failure ^41^^,^^42^.

Different risk factors have been described for the failure of miniscrews, including tobacco consumption, immediate charge of miniscrews and their design, among others. In this regard, Bayat *et al*. ^43^ showed that tobacco consumption alters gingival and bone micro vascularization. Smokers of more than 10 cigarettes per day have much higher failure rates (57.9%) compared to non-smokers (9.6%). The influence of immediate or late charge on miniscrew stability remains controversial ^(35, 42)^, although some studies have described that late charge after three weeks of healing time could be a factor in increasing miniscrew stability ^41^.

Longer screws with a wider diameter are considered more stable than shorter and narrower screws and present improved retention ^41^. Miniscrews with a diameter of 2 mm or more are more successful than those with a diameter of 1.5/1.8 mm, because they are not susceptible to fracture ^(35, 44)^. According to one study, the failure rate of miniscrews inserted at the infrazygomatic location was 16.4%. This high failure rate could be explained by the nature of the mobile gingiva at the miniscrew insertion site, as well as poor accessibility to this site during insertion and cleaning ^45^. In this regard, Uribe *et al*. ^44^ reported that miniscrews placed in the infrazygomatic region had a failure rate of 21.8%. This failure rate is higher than that reported by Liou *et al*. ^38^ who described a success rate of 100% for intraradicular miniscrews. Purely intrusive movements present lower failure rates compared to all other combinations of movements ^44^. However, Hsu *et al*. ^35^ found that infrazygomatic miniscrews have a low failure rate (6.35%). They evaluated 387 miniscrews in free mucosa and 385 in adhered mucosa for 6 months and after this time found that of the total of 722, 49 (6.37%) of the 387 devices placed in the free mucosa were lost and 25 (6.46%) of the 385 miniscrews placed in the adhered mucosa were lost, resulting in no statistically significant difference for both insertion sites. 

The failure rate on the right side (6.48%) was slightly higher than on the left (6.22%), but the difference was not statistically significant. Patients presenting screw failures were between 12 and 43 years old, with a mean age of 24.2 years, with no significant differences on comparing age groups ^35^.

The different results of Uribe *et al*. ^44^ and Hsu *et al*. ^35^ could be due to the fact that Uribe *et al*. ^44^ evaluated the miniscrews for around 13 months, while Hsu *et al*. ^35^ evaluated patients over 6 months, and thus, definitive conclusions cannot be made. In contrast, Ozdemir *et al*. ^46^ found that patients with an increased vertical skeletal pattern have reduced cortical bone thickness, which may affect the primary stability of miniscrews.

Therefore, it can be concluded that miniscrew placement should not fail if the appropriate equipment and miniscrews of 2 mm of diameter are used, the IZC insertion site is at the level between the first and second molars, the operator has the necessary knowledge, the patient is a non-smoker and good hygiene is maintained.

### Limitations

The limitations of miniscrews are few, one being the cost of miniscrews (which has reduced over time), and the age of the patient must also be taken into account since different studies have shown that there is greater failure in young patients due to the reduced height of the maxillary alveolar bone.

## CONCLUSIONS

It is concluded that the perforation of the miniscrew (stainless steel or titanium), the perforation of the maxillary sinus and the placement area (adhered or mobile mucosa) do not interfere with the success of miniscrews, which is only related to primary stability and insertion torque. It has been shown that the percentage of failure is lower in infrazygomatic compared to intraradicular miniscrews. For all these reasons, orthodontists can confidently and safely include infrazygomatic miniscrews in their orthodontic procedures.

## Financing Sources:

Self-financed
